# 

**Published:** 1880-11

**Authors:** 


### CONTENTS FOR NOVEM'R, 1880.

ORIGINAL COMMUNICATIONS.

Art. I.—Hip-Joint Operation. By G. Glanville Rusk, M. D. ....... 511
Art. II.—The Surgeons of Baltimore and their Achievements ....... 514
Art. III.—The Medical Schools of Baltimore. By Eugene F. Cordell, M. D. ....... 519
Art. IV.—Cancer Cures. Copied by Horace R. Mooney, M. D. ....... 523
Art. V.—Filling Roots of Teeth. By Samuel D. Rambo, D. D. S. ....... 524
Art. VI.—Treatment of Fracture of the Jaw, with Critical Remarks. By Thomas Brian Gunning, D. D. S. ....... 526

EDITORIAL DEPARTMENT.

Our Sesqui-Centennial ....... 535
Our Second Volume ....... 536
Opening of the Medical Schools ....... 537
The Equine Epizootic ....... 537
Tincture of Aconite Root and Veratrum Veride, in Puerperal Eclampsia ....... 537
Craniotomy ....... 538
"Minutes of the Twenty-fourth and Twenty-fifth Annual Meetings of the State Medical Society of Kentucky" ....... 538
Our Mortuary Reports ....... 538
Bound Volume of 1880 ....... 539

CORRESPONDENCE.

Pure Bath Rooms and Clean Water Closets ....... 539

BIBLIOGRAPHICAL DEPARTMENT.

Preadamites ....... 540
Hygiene and Sanative Measure for Chronic Catarrhal Inflammation of the Nose, Throat and Ears ....... 541
National Association for the Protection of the Insane ....... 541
Rocky Mountain Medical Review ....... 541
The Specialist and Intelligencer ....... 541
The Physicians' Visiting List ....... 542

Hymeneal, Necrological ....... 542

EXCERPTA.

Captain Eads' Ship Railway ....... 543
Learning to Smoke ....... 543
Influence of Electricity on Bacteria ....... 544
Prevention of Near-Sight in the Young ....... 545
Cæsarean Section on a Dwarf ....... 550
To Bleach Discolored Teeth ....... 551
Mercurial Amalgams ....... 552
Positive Sign of Pregnancy during the first three months ....... 553
Painless Operation for Ingrowing nails ....... 554
Beef Tea and its Value ....... 554
Should we Bandage after Labor, and Why? ....... 555

Mortuary Statistics ....... 558

